# Prenatal Exposure to Tobacco Smoke and Vaping Aerosols: Mechanisms Disrupting White-Matter Formation

**DOI:** 10.3390/toxics13121071

**Published:** 2025-12-12

**Authors:** Sebastián Beltran-Castillo, Juan Pablo Espinoza, Michelle Grambs

**Affiliations:** 1 Centro Integrativo de Biología y Química Aplicada (CIBQA), Universidad Bernardo O’Higgins, Santiago 8370993, Chile; 2 Doctorado en Ciencias Mención Materiales Funcionales, Facultad de Ciencias de la Salud, Universidad Bernardo O’Higgins, Santiago 8370993, Chile; 3 Medical Faculty Heidelberg, Ruprecht-Karls-University Heidelberg, 69117 Heidelberg, Germany

**Keywords:** prenatal exposure, white-matter development, oligodendrocyte differentiation, neurodevelopmental toxicity

## Abstract

White-matter development during fetal life represents one of the most vulnerable processes to environmental disruption, as it relies on the precisely timed proliferation, migration, and differentiation of oligodendrocyte lineage cells. Among environmental threats, exposure to toxic compounds contained in tobacco smoke and vaping aerosols represents a major yet preventable risk during pregnancy. Despite growing awareness, tobacco smoking remains widespread, and a substantial proportion of the population—including pregnant women—continues to perceive electronic nicotine delivery systems (ENDS) as less harmful, a misconception that contributes to persistent prenatal exposure. These products expose the fetus to numerous substances that readily cross the placenta and reach the developing brain, including compounds with endocrine-disrupting activity, where they interfere with white-matter development. Epidemiological and neuroimaging studies consistently reveal microstructural alterations in white matter that correlate with long-term cognitive and behavioral impairments in offspring exposed in utero. These alterations may arise from both nicotine-specific pathways and the actions of other toxicants in cigarette smoke and ENDS aerosols that cross the placenta and disrupt white-matter emergence and maturation. Preclinical research provides mechanistic insight: nicotine acts directly on nicotinic acetylcholine receptors (nAChRs) in oligodendrocyte precursor cells, disrupting calcium signaling and differentiation, while additional constituents of smoke and vaping aerosols also affect astrocyte and microglial function and disturb the extracellular milieu required for proper myelination.

## 1. Introduction

For smokers, tobacco use is often associated with relaxation and pleasure. However, both active and passive smokers are exposed to more than 7000 chemicals, many of them addictive, such as nicotine, and highly toxic, such as carbon monoxide, formaldehyde, acrolein, heavy metals (e.g., cadmium and arsenic), and nitrosamines. Electronic nicotine delivery systems (ENDS), although often misperceived as less harmful, still expose users to vaping aerosols contain nicotine and solvents such as propylene glycol and vegetable glycerin, as well as flavoring compounds which, when heated, undergo thermal decomposition into highly toxic aldehydes, including formaldehyde, acetaldehyde, and acrolein [1]. ENDS also expose users to metals released from the heating coil, such as nickel, chromium, iron, tin, and copper [2]. Many of these substances are teratogenic, making the developing fetus in pregnant smokers or passive smokers—particularly its brain—highly vulnerable to these chemicals, which can impair brain development.

Most studies have traditionally emphasized neuronal alterations associated with prenatal cigarette exposure. However, evidence shows that glial cells such as oligodendrocytes, the cells that produce myelin in the central nervous system, and their progenitors, the oligodendrocyte precursor cells (OPCs), are also susceptible to toxicants from cigarettes and ENDS, which readily cross the placenta and reach the developing brain. The intrinsic vulnerability of the oligodendroglial lineage to cigarette and ENDS toxicants is exemplified not only by the expression of several subtypes of nicotinic acetylcholine receptors (nAChRs), through which nicotine can act directly, but also by alterations in the surrounding glial microenvironment caused by other toxicants present in cigarette smoke and ENDS aerosols, which can impair glial function and the conditions required for proper myelination. Consequently, prenatal exposure to cigarette smoke and substances from ENDS may interfere with the development of white matter during fetal life, as the developing brain undergoes rapid waves of oligodendrogenesis and myelination, stages that require precise temporal coordination between cell proliferation, migration, and differentiation. Disruption of these processes may irreversibly alter axon–glia interactions, impair myelin formation, affect white-matter integrity, and compromise neural circuit maturation, potentially leading to persistent abnormalities.

In this review, we integrate epidemiological, neuroimaging, and experimental evidence to describe the mechanisms by which prenatal exposure to components of cigarette smoke and vaping aerosols may affect oligodendroglial development, myelin formation, and white-matter organization, given that a critical window for white-matter development occurs during the prenatal period. It is also important to note that many studies refer to “nicotine exposure” when this actually reflects exposure to whole cigarette smoke, whose complex toxicant mixture differs markedly from pure nicotine, making direct interpretation of nicotine-specific effects more difficult. In addition, the addictiveness of pure nicotine in isolation remains debated, as many addictive properties attributed to “nicotine” in the literature actually arise from the complex toxicant mixture present in whole cigarette smoke. We first summarize findings from human studies showing that associate alterations in white-matter structure and function with prenatal exposure to cigarette smoke or vaping aerosols. We then focus on preclinical research exploring the underlying mechanisms for white-matter impairment, including mechanisms mediated by nicotine as well as those driven by other toxicants that cross the placenta and may disrupt glial maturation and myelination. All these findings underscore the importance of maintaining and reinforcing prevention efforts to reduce prenatal exposure to tobacco and vaping products.

## 2. The Prenatal Period as a Critical Developmental Window for White-Matter Formation

Approximately half of the brain’s volume is composed of white matter, a structure consisting of bundles of nerve fibers, most of them myelinated by oligodendrocytes, and accompanied by astrocytes, microglia, and blood vessels that provide metabolic and structural support [3]. The formation of white matter depends on a tightly orchestrated sequence of developmental events that begin early in gestation and continue throughout life, providing the foundation for long-range axonal connectivity.

During the second and third trimesters of gestation, major white-matter pathways undergo rapid development in the human brain [4,5,6], providing the structural basis for later functional brain integration. Evidence from in utero diffusion magnetic resonance imaging (dMRI), an advanced magnetic resonance imaging (MRI) technique that characterizes the microstructural organization of brain tissue by measuring the diffusion of water molecules along neural fibers, has revealed that major white-matter tracts follow specific and dynamic maturational trajectories before birth [6], and this growth continues markedly after birth, representing one of the most active periods of white-matter development that extends up to the first three years of life [7].

Consistent with the notion that long-range connectivity begins to emerge during this period, early corticocortical and thalamocortical interactions are also shaped by the subplate zone, a transient compartment present between approximately 15 and 24 weeks of gestation that orchestrates the formation of the horizontal and vertical matrix of the developing frontal cortex and establishes the initial scaffold for long-range projections [8]. The subplate operates within a metabolically demanding and hormone-regulated environment, making it highly sensitive to hypoxic, inflammatory and endocrine-disrupting exposures [9]. In this context, prenatal exposure to cigarette smoke and ENDS aerosols may disrupt axonal growth, synaptic activity, and differentiation within the subplate, potentially altering the early architectural template on which major white-matter pathways are built.

**Figure 1:**
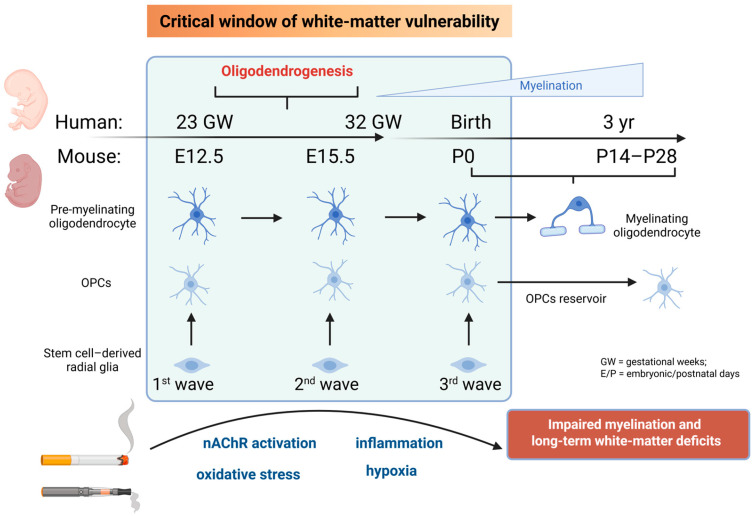
Developmental timeline of oligodendrogenesis in humans and mice highlighting the critical window of vulnerability (23–32 gestational weeks; E12.5–E15.5 in mice). During this period, late oligodendrocyte progenitors are highly sensitive to inflammation, oxidative stress, and hypoxia, leading to impaired myelination and long-term white-matter deficits.

Overall, this is a critical period for an adequate emergence of white matter, and deviations from the normal developmental pattern, such as exerted by maternal stress, or exposure to cigarette toxins may therefore have profound consequences for neural efficiency and higher-order functions in newborns [7,10,11] (Figure 1). In fact, immature white matter, such as that observed in premature newborns, remains highly vulnerable to hypoxic or metabolic insults [12], highlighting the importance of proper white-matter formation in fetal life to ensure efficient brain connectivity after birth.

The existence of a critical window for white-matter formation is also supported by evidence from preclinical models and postmortem human brain examination regarding how oligodendrocyte lineage emergence. In mice, oligodendrocytes arise after neurogenesis from neural stem cell-derived radial glia, which give rise to OPCs that proliferate and migrate across the CNS in successive waves—at embryonic days E12.5 and E15.5 in the spinal cord and brain, and around postnatal day 0 (P0) in the brain [13,14]. Some OPCs undergo partial differentiation into pre-myelinating oligodendrocytes, whereas others persist as OPCs into adulthood, serving as a cellular reservoir. Finally, pre-myelinating oligodendrocytes mature into fully myelinating cells that form compact myelin sheaths around axons with a peak between 2–4 weeks after birth [15,16]. In other words, these data indicate that during the prenatal period, white matter remains immature. Similarly, postmortem studies in the human brain have revealed that between 23 and 32 weeks of gestation, late OPCs (NG2^+^/O4^+^) predominate [17], coinciding with a critical clinical window for periventricular white-matter injury (PVL), where white matter is particularly susceptible to insults such as oxidative stress, hypoxia, and inflammation [18], just before the onset of extensive myelination that occurs later after 32 weeks [17].

## 3. Global Epidemiology of Prenatal Tobacco Exposure

Prenatal tobacco exposure remains a major public health concern, especially in low- and middle-income countries where the rate of smoking is still relatively high [19,20]. For example, in Portugal, 14.6% of women continued smoking at the time of delivery, and 49.8% of non-smokers were exposed to secondhand smoke [19]. In the area surrounding Cape Town, South Africa, 58.3% of pregnant women smoked during pregnancy [21], and similarly, another study conducted in nearby communities from the same region found that 32% of pregnant women were active smokers, as confirmed by urinary cotinine testing [22].

Fortunately, in high-income countries such as the United States, the prevalence of cigarette smoking during pregnancy has decreased since the early 2000s [23,24]. By 2016, 7.2% of women who gave birth reported smoking during pregnancy, with rates exceeding 8.2% among women aged 15–29 years [25] whereas by 2021, had declined to 4.6% and 4.5%, respectively [24]. However, the percentage of pregnant smokers remained highest among non-Hispanic American, Indian or Alaska Native women and among pregnant women from West Virginia, where 12.7% and 18,2%, respectively, reported smoking during pregnancy in 2021 [24]. Moreover, lower educational level has been significantly associated with higher smoking prevalence, greater exposure to secondhand smoke and lower smoking cessation rates [19,26].

These disparities in prevalence reflect differences in tobacco control policies, socioeconomic factors, and cultural attitudes toward smoking, but also a gradual replacement of tobacco with ENDS. In the United States, approximately 6.8% of pregnant women use ENDS [27]. Moreover, 24.4% of women who used e-cigarettes before pregnancy continued vaping during gestation [28]. Globally, the prevalence of ENDS use during pregnancy is estimated to range from 2.2% to 4.8% [29]. Interestingly, 64.27% of participants viewed e-cigarettes as less harmful than tobacco cigarettes [30], supporting the observation that ENDS are widely perceived as less harmful, including among pregnant women—a misconception with significant implications for prenatal exposure. Such misperceptions highlight the need for better public health communication to correct misunderstandings about the safety of ENDS during pregnancy.

## 4. Neurodevelopmental Effects of Exposure on White Matter

Exposure to tobacco smoke, ENDs and its toxic constituents is not innocuous for the fetus. Even brief exposure—such as a mother smoking a single cigarette—produces measurable hemodynamic changes, increasing placental vascular resistance from the fetal side [31], which in turn impairs gas exchange and promotes fetal hypoxia. Several toxicants including carbon monoxide, aldehydes, nitrosamines, metals and nicotine, can reach the fetal circulation. Nicotine readily crosses the placenta and the fetal blood–brain barrier, where its slower metabolism may result in prolonged fetal exposure [32,33], while other toxicants contribute through mechanisms such as hypoxia, oxidative stress, inflammation, and endocrine disruption. Together, these placental and neurotoxic effects heighten the vulnerability of the developing brain, and white matter is no exception.

Therefore, it is unlikely that prenatal exposure to tobacco smoke or ENDS aerosols would have no consequences for the emergence and maturation of white matter. In the following paragraph, we summarize the human neuroimaging evidence that supports the impact of prenatal tobacco exposure on white matter, emphasizing alterations in its microstructure and associated behavioral and cognitive outcomes. Most human neuroimaging studies available to date refer specifically to prenatal exposure to combustible tobacco smoke; thus, the findings in this section predominantly reflect maternal smoking. Evidence on ENDS use during pregnancy exists, but no neuroimaging studies have yet examined its impact on fetal or infant white-matter development. Later, in Section 5, we review mechanistic and experimental findings from animal models that help clarify the cellular and molecular pathways through which nicotine and other toxicants from tobacco smoke and ENDS aerosols may disrupt white-matter development.

### 4.1. Human Imaging Studies Reveal Alteration in White Matter

Prenatal tobacco exposure has been evaluated in relation to brain morphometric measures in offspring using MRI. Findings revealed that children in late childhood who were exposed in utero not only exhibit smaller caudate nucleus volumes, but also lower gray–white matter contrast across widespread regions of the parietal, temporal, and frontal lobes [34], which in turn could reflect myelination deficits, but may also result from increased gray matter signal intensity. However, there is others evidence that reveal consistent abnormalities in white-matter microstructure following prenatal tobacco exposure in children. Toddlers aged 2–3 years with prenatal tobacco exposure show lower mean diffusivity in the splenium of the corpus callosum, suggesting greater fiber density, premature myelination, or abnormal axonal compactness [35]. Similarly, in a cohort of 410 participants aged 8–12 years, lower fractional anisotropy (FA) and higher radial diffusivity (RD), two MRI-derived measures of white-matter organization and myelin integrity, were detected in major projection tracts, particularly among those exposed during the second trimester, when white matter exhibits the greatest vulnerability [36]. These findings indicate disrupted axonal organization and myelin integrity, suggesting that prenatal exposure to tobacco toxins interferes with white-matter maturation during critical windows of neurodevelopment.

Other data reveal the long-term impact of prenatal exposure on white matter. Adolescents with prenatal tobacco exposure show not only increased FA in anterior cortical white matter and within the internal capsule, particularly in regions containing auditory thalamocortical and corticofugal fibers [37,38], but also decreased FA in the supplementary motor area and premotor cortex [39]. Despite opposite FA directions, both studies converge on demonstrating atypical microstructural development of white matter, likely reflecting aberrant axonal coherence or altered myelin compactness. Taken together, these findings suggest that prenatal tobacco exposure induces persistent and region-specific alterations in white-matter integrity, which may underlie long-term differences in cognitive and sensory processing.

### 4.2. Neuroimaging and Its Correlation with Behavioral and Cognitive Impairment

Neuroimaging evidence suggests that cognitive and behavioral outcomes associated with prenatal tobacco exposure may in part depend on white matter integrity. Microstructural abnormalities in major projection tracts suggesting atypical axonal organization or myelin compactness [36] are consistent with impairing the efficiency of neural communication between cortical and subcortical region able to affect the attention, executive function and emotional regulation. Functional MRI studies in young adults prenatally exposed to maternal smoking revealed hypoactivation in the anterior cingulate and inferior frontal gyri during inhibitory control tasks, together with reduced inferior frontal volume [40], indirectly pointing to compromised fronto-cingulate connectivity supported by white-matter tracts. In addition, increase in FA in the posterior limb of the left internal capsule correlates with longer reaction time during an auditory attention task, suggesting that tobacco-related microstructural changes may compromise the efficiency of auditory and attentional pathways [37,41], since these children have been reported in many works with deficit in attention associate with auditory and visual attention tasks [38]. On the other hand, the alterations detected by MRI in gray–white matter contrast (GWC) across the frontal, temporal, and parietal lobes [34], are consistent with previous reports indicating that maternal tobacco use during pregnancy is associated with language processing difficulties [42] and poorer episodic memory [43,44] in exposed offspring.

The correlations described above reflect postnatal alterations in white-matter microstructure and connectivity that are compatible with early disturbances in the fetal subplate zone. Perturbations to subplate-mediated axonal guidance and early myelination may leave long-lasting imprints on the organization and efficiency of the tracts later reflected in FA, RD, GWC, or functional MRI measures. In this sense, the cognitive and sensory deficits described in children and adolescents with prenatal tobacco exposure may partly reflect the downstream consequences of altered subplate-dependent circuit formation during mid-gestation.

An additional developmental dimension involves sex differences in brain connectivity and lateralization, which depend on sexually dimorphic patterns of white-matter myelination, particularly within commissural pathways such as the corpus callosum. MRI studies indicate that males and females differ in global myelination levels, inter- versus intra-hemispheric connectivity profiles, and in the timing of regional myelin maturation [45,46,47]. Notably, some of these dimorphisms are already detectable in early childhood, including the preschool years [48]. Disorders that disrupt white-matter integrity can modify typical sex-dependent patterns of callosal structure and connectivity. For example, in first-episode, drug-naïve schizophrenia, widespread reductions in FA—including within the corpus callosum—show sex-specific associations with clinical symptomatology, indicating that alterations in white matter may reorganize or differentially modulate male–female patterns of interhemispheric connectivity [49]. These observations suggest that any factors affecting the emergence and maturation of white matter—such as that induced by prenatal exposure to tobacco toxicants—may disrupt the timing and coordination of sexually dimorphic myelination trajectories. Such perturbations could, in turn, compromise the establishment of typical sex-dependent lateralized networks, generating developmental imbalances that increase vulnerability during prenatal and early postnatal life.

### 4.3. Prenatal Exposure and Susceptibility to Demyelinating Disease

A higher occurrence of demyelinating diseases has been consistently associated with tobacco exposure. Epidemiological data indicate that current smokers have a significantly higher risk of developing multiple sclerosis (MS) compared to never-smokers, although the magnitude of this association and its impact on disease progression vary across studies [50]. Smokers also show an increased likelihood of developing primary progressive MS (characterized by a steady accumulation of neurological disability from disease onset) and a faster progression from relapsing–remitting MS (characterized by clearly defined episodes of neurological dysfunction followed by periods of partial or complete recovery) to secondary progressive forms, in which neurological damage accumulates gradually and irreversibly [51,52].

On the other hand, a few epidemiological studies have suggested a correlation between perinatal exposure to tobacco components and an increased incidence of MS. One of these was a population-based case–control study conducted in France, which evaluated 129 cases of children diagnosed with MS before age 16. Using parental questionnaires and conditional logistic regression, the researchers determined that parental smoking may double the risk of developing MS [53]. Similarly, a study performed across nine federal hospitals in the state of Washington, United States, also identified an association between prenatal exposure to tobacco components and MS [54]. In addition, a large Danish nationwide cohort including all pregnant women from 1991 to 2018 revealed that offspring born to smoking mothers had a 38% higher risk of developing MS compared with those born to non-smokers [55]. However, these results contrast with those obtained from a Swedish cohort study, in which the researchers did not find a significant association between maternal smoking during pregnancy and MS risk in offspring [56]. These findings suggest that prenatal exposure alone may not be sufficient to cause demyelinating diseases such as MS but may increase the risk, likely requiring additional genetic or environmental factors. Nevertheless, determining whether exposure to tobacco components during prenatal life contributes to the emergence of demyelinating diseases in adulthood remains a challenging question.

In the following sections, we summarize evidence from preclinical models demonstrating that such prenatal exposure to tobacco substances (or vaping aerosol) can alter oligodendrocyte lineages, thereby providing mechanistic insight into how early-life toxicant exposure might predispose to white-matter vulnerability later in life.

## 5. Mechanistic Insights into Myelin Vulnerability Induced by Prenatal Tobacco Exposure

The epidemiological evidence described in Section 3 links prenatal tobacco exposure to alterations in early white-matter development. The following section summarizes current insights into the cellular and molecular mechanisms underlying these effects. The mechanistic literature on prenatal exposure to tobacco smoke and ENDS aerosols is historically uneven, with a disproportionately large body of work focused on nicotine—often using postnatal rodent models—relative to other toxicants. Although several non-nicotine toxicants (e.g., carbon monoxide, aldehydes, nitrosamines, metals) have broader or more direct prenatal evidence in general, mechanistic studies specifically addressing their impact on white-matter development remain comparatively scarce and fragmented. This imbalance in the available literature reflects historical research patterns rather than a greater biological relevance of nicotine, and therefore the mechanisms summarized in this section should be interpreted within a broader multipollutant context.

For this reason, Section 5 first summarizes the more extensively characterized nicotine-mediated pathways and then integrates the available evidence for non-nicotine toxicants, providing a balanced, multipollutant framework rather than a nicotine-centered perspective.

### 5.1. Nicotinic Signaling and Oligodendrocyte Lineage Vulnerability

Nicotine is the best-characterized component of tobacco smoke and ENDS aerosols in relation to glial development, with mechanistic evidence derived from both prenatal and postnatal exposure models. Prenatal studies demonstrate disruptions in oligodendrocyte maturation and early myelination, while postnatal models have contributed complementary insight into the intracellular pathways and receptor-mediated mechanisms affected by nicotine. Because this body of evidence is unevenly distributed across developmental stages, the mechanisms summarized below integrate findings from both prenatal and postnatal studies and are considered within a broader multipollutant framework that also includes additional toxicants relevant to prenatal white-matter vulnerability (discuss later).

Nicotine is an agonist that acts on nicotinic acetylcholine receptors (nAChRs), which are pentameric ligand-gated ion channels composed of different combinations of α and/or β subunits [57]. The ionic permeability of nAChRs depends on their subunit composition, being permeable to small monovalent and divalent cations such as Na+, K+, Ca2+ [58]. RT-PCR and immunocytochemical analyses of O2A/OPCs isolated from the corpus callosum of 7-day old rats revealed the presence of multiple nAChR subunits, including α3, α4, α5, α7, β2, and β4 in OPCs [59]. Because these are the cells that differentiate and mature into myelinating oligodendrocytes, their exposure to nicotine during critical white-matter developmental windows may disrupt the normal molecular program governing white-matter establishment [3].

Electrophysiological and calcium-imaging analyses demonstrated that nAChR subunits expressed in O2A/OPCs isolated from the corpus callosum of 7-day-old rats are functional, as at least two-thirds of the cells responded to nicotine stimulation with a rapid increase in intracellular calcium levels [59]. The response was sensitive to α4β2 nAChR antagonist dihydro-β-erythroidine (DHβE) and to the voltage-gated calcium-channel blocker nifedipine, indicating that both receptor activation and secondary calcium influx contribute to the signal. In a subset of the nicotine- responsive cells, calcium response occurred in an oscillatory pattern with intervals of 20–30 s that gradually decreased in amplitude, suggesting that nAChRs can reopen from desensitized states and sustain rhythmic calcium signaling. Complementary evidence from hippocampal slices of mice revealed that NG2- cells, another population of OPCs, express functional α7- containing nAChRs during the second postnatal week [60]. Activation of these receptors produced calcium influx that was greatly enhanced by the positive allosteric modulator PNU-120596, confirming that α7-nAChRs in OPCs are highly Ca^2+^-permeable but also prone to desensitization by nanomolar concentrations of nicotine. Together, these findings demonstrate that nicotinic signaling in OPCs can evoke both α4β2- and α7-dependent calcium responses that may be part of the regulatory mechanism controlling key calcium-dependent processes such as proliferation and differentiation [61,62,63,64,65]. Consequently, prenatal nicotine exposure in experimental models may interfere with the normal timing and pattern of oligodendrocyte maturation.

Sensitivity to nicotine is also evident when prolonged exposure to nicotine vapor, mimicking e-cigarette use, promotes oligodendrocyte differentiation in adult male rats. Specifically, nicotine increased OLIG2 expression in the ventral tegmental area (VTA), together with the upregulation of the histone demethylase enzyme Kdm6b, brain-derived neurotrophic factor (Bdnf), and tropomyosin receptor kinase B (TrkB), all genes involved in oligodendrogenesis. These findings suggest that nicotine delivered through vaping may directly or indirectly activate molecular programs that promote oligodendrocyte differentiation and myelin remodeling [66].

Regarding the implications of prenatal nicotine exposure directly affecting OPC maturation, the available evidence remains limited, and further experiments are needed to elucidate the specific mechanisms involved. However, some data show that prenatal nicotine exposure in Sprague–Dawley rats (3 mg kg^−1^ day^−1^ from gestational days 4–18) produces long-lasting alterations in the expression of multiple myelin proteins, lipid-related enzymes and myelin- associated transcription and trophic factors in the offspring [67]. At postnatal days 35–36, juvenile rats displayed region- and sex-specific changes in the expression of myelin-related genes and proteins within limbic regions such as prefrontal cortex (PFC), caudate–putamen (CPu), and nucleus accumbens (NAc). In the PFC of male offspring, mRNA levels of myelin basic protein (Mbp), myelin-associated oligodendrocytic basic protein (Mobp), proteolipid protein 1 (Plp1), myelin-associated glycoprotein (Mag), gap junction membrane channel protein epsilon 1 (Gje1), gap junction protein alpha 12, 47 kDa (Gjc2), Claudin11 (Cldn11) were significantly increased, whereas in female offspring, all genes except Gjc2 were significantly downregulated [67]. Consistently, lipid- related enzimes such as UDP glycosyltransferase 8 (Ugt8) and aspartoacylase (Aspa) were upregulated only in males. Similarly, transcription factors essential for oligodendrocyte differentiation such as OLG transcription factor-1 (Olig1), Olig2, and Olig3 and SRY box 10 (Sox10) were increase in males, and another such Olig3, Sox10 and NK6 homeobox 2 (Nkx6-2) decreased in female [67].

In the CPu, prenatal nicotine exposure upregulated Cldn11, Myelin and lymphocyte protein (Mal), Gjc2, Mbp, Mobp, Plp1, 2′,3′-cyclic nucleotide 3′-phosphodiesterase (Cnp), Mag, myelin oligodendrocyte glycoprotein (Mog), Ugt8 and Aspa exclusively in male rats. In the NAc, Mbp, Mobp, Plp1, Cnp, Mog, Gjb1 and Ugt8 were upregulated in males, while only Mbp and Plp1 mRNA levels expression were increased in female [67]. With respect to myelin transcription factors, gestational nicotine exposure increased the expression of Olig1, Olig2, Sox8, Sox9, Sox10, Nkx2-2, and Nkx6-2, in male offspring, whereas in females, only Sox9 expression was significantly upregulated [67]. Together, these findings demonstrate that gestational nicotine exposure alters myelin-related transcriptional programs in a sex- and region-dependent manner, with a long-lasting impact.

Evidence obtained in zebrafish further supports the vulnerability of myelin development to nicotine in progeny, even when maternal exposure to nicotine begins before pregnancy. Adult female zebrafish exposed to nicotine (1–30 μM) for four months before mating produced offspring with altered developmental trajectories of myelin gene expression. Specifically, the mRNA levels of major myelin proteins were downregulated in early larvae (4 dpf) but upregulated at later stages (14 dpf), and transcriptional regulators followed a similar biphasic pattern. These findings suggest that maternal nicotine exposure—even prior to gestation—can indirectly reprogram myelin-related gene networks across developmental stages, potentially disrupting the timing of oligodendrocyte maturation and myelin formation in the offspring [68]. In addition, these results likely reflect systemic or glial-dependent processes in the mother—such as inflammatory or epigenetic reprogramming in mother—initiated by nicotine exposure before conception, which persist into embryonic development and compromise white-matter integrity in the offspring.

**Figure 2:**
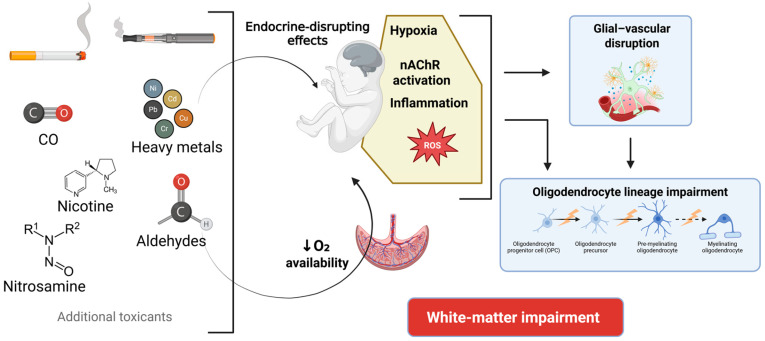
Mechanistic model linking prenatal exposure to cigarette smoke and ENDS with fetal white-matter impairment. Combustion-derived toxicants from cigarette smoke (e.g., carbon monoxide, nitrosamines, aldehydes, heavy metals) and heating-derived toxicants from ENDS aerosols (e.g., nicotine, aldehydes formed by thermal decomposition, and metals from the heating coil) reach the fetal brain and trigger distinct but partially overlapping biological responses. These include hypoxia, oxidative stress, inflammation, endocrine-disrupting effects which interfere with hormone-regulated myelination, and nAChR-mediated signaling. These insults act directly on oligodendrocyte-lineage cells or indirectly through astroglial, microglial, and endothelial activation, leading to glial–vascular disruption and impaired oligodendrocyte differentiation and myelin formation. Collectively, these converging pathways result in white-matter impairment during prenatal brain development. Most mechanistic insights summarized in this figure derive from preclinical studies in animal models, where individual toxicants or controlled mixtures can be examined directly. In humans, the biological effects will depend on the specific combination, concentration, and timing of exposure.

Overall, these findings highlight that nicotine can influence white-matter development through both direct and indirect mechanisms (Figure 2). While prenatal paradigms encompass cell-autonomous actions of nicotine on the oligodendrocyte lineage—mediated by nAChRs expressed in OPCs—they also involve non-cell-autonomous processes driven by maternal–placental physiology, endocrine alterations, or neuroimmune activation. The sex- and region-specific myelin gene changes reported in rodents [67] and the biphasic expression trajectories observed in zebrafish following preconception nicotine exposure [68] likely represent the combined outcome of these pathways. This dual-path framework suggests that, beyond receptor-mediated effects, glial-dependent mechanisms—particularly involving astrocytes and microglia—may play a crucial role in shaping the white-matter deficits observed after developmental nicotine exposure. In this context, nicotine-induced inflammatory and oxidative processes may alter astrocyte and microglial function, conditioning the extracellular environment and disrupting the normal trajectory by which OPCs mature into myelinating oligodendrocytes. This possibility is explored in the following section.

### 5.2. Indirect Glial-Mediated Mechanisms: Nicotine-Mediated Astrocyte and Microglial Dysfunction Affecting White-Matter Development

Nicotine does not act exclusively on neurons or oligodendrocytes; it can also act on astrocytes and microglia, as both cell types express nAChRs. The data and their interpretation regarding the effects of nicotine on white-matter development through astrocytic and/or microglial mechanisms are complex, mainly due to the simultaneous activation of multiple molecular signals and the interactions among glial cell populations. However, these effects likely involve alterations in glial communication and support functions that, as a secondary consequence of nicotine exposure, modify the local environment and ultimately influence normal white-matter development (Figure 2).

#### 5.2.1. Astrocytes

Multiple nAChR subunits have been detected in astrocytes, including β4 in astrocytes from mouse [69] and α7, α4, and β2 in astrocytes from rat [70]. However, only homomeric α7 nAChRs have been reported such as fully functional, promoting increases in intracellular Ca^2+^ and calcium release from intracellular stores [71,72].

Acute nicotine exerts a protective effect on astrocytes by preventing apoptosis induced by hydrogen peroxide and downregulation of glial cell-derived neurotrophic factor (GDNF) downregulation (which promote survival), also reducing its activation in the substancia nigra pars compacta [73]. Nevertheless, chronic exposure induces structural remodeling, including elongation of fine and proximal processes, increased cell volume, and elevated Ca^2+^ signaling [73]. Such morphological and functional adaptations, while not overtly cytotoxic, are considered maladaptive, as they may enhance synaptic hyperexcitability and sensitization mechanisms underlying nicotine dependence.

Regarding prenatal exposure, it has been shown that nicotine modifies astrocyte reactivity in the developing brain. In guinea pigs, it increases the density of Glial Fibrillary Acidic Protein (GFAP)-positive astrocytes in regions such as the retrosplenial cortex (RSg) and hippocampal CA1 [74], and in the cerebellum and hippocampus of rats [75]. This upregulation of GFAP suggests an enhancement in astrocytic reactivity induced by nicotine which may alter environment where white-matter development occurs. However, other studies [76,77] did not report significant changes in GFAP expression under similar experimental conditions, suggesting that the response to prenatal nicotine may depend on species or dosage.

Interestingly, prenatal nicotine exposure also promotes increases on the opening of connexin-43 (Cx43) hemichannels in mice astrocyte at 8 weeks of age, leading to excessive ATP and glutamate release [78]. Since ATP can stimulate OPC migration while inhibiting their proliferation and promoting oligodendrocyte differentiation, a potential dysregulation of this trophic communication between astrocytes and oligodendrocyte lineage cells could impair the normal timing of white-matter formation.

On the other hand, prenatal exposure to ENDS components shows reduced expression of tight-junction proteins ZO-1 and claudin-5, disrupting the blood–brain barrier [79], an effect also observed by in situ brain perfusion with nicotine [80]. These effects increase vascular permeability to peripheral substances [81], thereby exacerbating exposure to toxins with potential to impair white matter formation.

#### 5.2.2. Microglia

Microglia express α7 nAChRs, whose activation attenuates neuroinflammatory responses by reducing TNF-α release [82,83]. These receptors are localized primarily in mitochondria rather than at the plasma membrane of microglia, where they modulate mitochondrial ATP production rather than acting as ionotropic channels [84]. This mitochondrial localization can imply a metabolic role in maintaining energy homeostasis and inflammatory tone. This mitochondrial localization suggests a metabolic role for α7 nAChRs in maintaining energy homeostasis and inflammatory tone. Consequently, prenatal nicotine exposure could alter microglial mitochondrial function through these intracellular α7 nAChRs, impairing microglial metabolism and immune responsiveness during prenatal development, and thereby disrupting the normal pattern of white-matter formation.

In rats, maternal nicotine exposure via drinking water during gestation increases the anti-inflammatory M2 microglial phenotype in the hippocampus, accompanied by decreased IL-4 (a pro-inflammatory cytokine) and increased IL-1β (an anti-inflammatory cytokine) in the offspring. Nevertheless, these rats exhibit anxiety-like behavior and reduced NeuroD1 expression—a key protein for neuronal maturation and survival—indicating functional impairment [85]. The authors interpreted this anti-inflammatory shift as a compensatory response attempting to counteract nicotine-induced injury rather than a genuinely beneficial adaptation. However, microglia polarized toward the M2 phenotype can also drive oligodendrocyte differentiation [86]; therefore, a persistent or premature M2 bias could disturb developmental timing. Indeed, physiological M1-to-M2 transitions are essential for the developing brain: early M1-type activation supports normal neurogenesis and oligodendrogenesis, while its inhibition disrupts paracrine cues necessary for neuronal and glial maturation [87]. Thus, prenatal nicotine exposure, by shifting or prolonging M2 polarity, may impair the pro-developmental inflammatory signaling window required for proper white-matter development.

### 5.3. Other Tobacco and ENDS Toxicants Implicated in White-Matter Vulnerability

Although nicotine remains the most extensively characterized toxicant with respect to glial lineage biology, several other components of cigarette smoke and ENDS aerosols have stronger or more direct evidence for prenatal toxicity overall. However, mechanistic studies specifically linking these toxicants to oligodendrocyte maturation and myelination remain fewer in number. Since tobacco smoke and vaping aerosols are complex chemical mixtures, multiple non-nicotine constituents also contribute to white-matter vulnerability during prenatal development. Among them, carbon monoxide (CO), which competes with oxygen for hemoglobin binding, can induce maternal and fetal hypoxemia. Experimental evidence supports the high sensitivity of myelination to such hypoxic conditions. In a classical study, kittens delivered by cesarean section or at full term after acute maternal CO intoxication (0.2–0.3% for 2.5 h) showed extensive white-matter compromise [88]. Similarly, in rats, prenatal exposure to low CO concentrations (75–150 ppm from gestational day 0 to 20)—producing maternal HbCO levels comparable to those found in human smokers—led to subtle but persistent reductions in myelin sheath thickness within sciatic nerve fibers of male offspring [89]. These deficits appeared after the main developmental spurt of myelination and were not accompanied by changes in axon caliber or overt motor dysfunction. Interestingly, the myelin deficit became evident only after the main myelination surge, suggesting that prenatal CO exposure does not prevent the initiation of myelin formation but rather impairs its later maturation and stabilization [89], resulting in hypomyelination.

Other substances, such as aldehydes, affect the redox buffering and detoxification capacity of oligodendroglia [90], increasing their vulnerability to oxidative stress [91] and reducing oligodendrocyte differentiation [92]. Notably, the solvents commonly used in e-liquids (propylene glycol and vegetable glycerin) thermally decompose into reactive aldehydes such as formaldehyde, acetaldehyde, and acrolein [93,94], thereby expanding the potential sources of carbonyl-induced oxidative injury during prenatal or adolescent vaping exposure.

Heavy metals such as Cd, Pb, Ni, may accumulate in the brain [95] and promote oxidative stress that affects myelination [96]. Experiments conducted on fetuses and embryos have shown that brief exposure to cadmium (25–100 μM) alters oligodendrocyte stability and induces mitochondria-dependent apoptotic death in OPCs [97]. Similarly, prenatal exposure of rats from gestational day 5 via gavage with a mixture of As, Cd, and Pb has toxic effects, including a reduction in the area of intact myelinated fibers and an increase in vacuolated axons, particularly in the corpus callosum [98]. Pb at 1 μM inhibits OPCs differentiation via decreasing the expression of Olig 2, CNPase proteins in vitro [99].

Beyond their oxidative and cytotoxic effects, heavy metals such Cd and Pb also exert endocrine-disrupting actions relevant to white-matter development (Figure 2). Both function as metalloestrogens by binding to and activating estrogen receptors, eliciting estrogen-like transcriptional responses in vitro and in vivo [100,101,102]. This mechanism is highly relevant because estrogens are known positive regulators of oligodendrocyte differentiation, myelin formation, and the survival of premyelinating oligodendrocytes [103].

Nitrosamines contained in tobacco and ENDS liquids, such as 4-(methylnitrosamino)-1-(3-pyridyl)-1-butanone (NNK), are not only carcinogenic but, when exposure occurs prenatally in rats, also induce changes in immature and mature oligodendroglial genes that regulate OPC proliferation and maturation, while reducing MBP expression in oligodendrocytes [104]. Furthermore, ENDS devices are a source of metal exposure, as their heating coils and metallic components often leach elements such as Ni, Cr, Cu, Pb, and Sb into the aerosol [2]. Laboratory analyses of pod-type and disposable devices demonstrate that metal concentrations in the aerosol increase with repeated puffs and device age, sometimes exceeding risk thresholds for cancer and neurotoxicity [105,106]. These metals are known to accumulate in the brain and glial cells and have the potential to disrupt calcium signaling, oxidative balance, and oligodendrocyte function.

Collectively, these diverse toxicants, through converging mechanisms involving hypoxia, oxidative stress, glial metabolic disruption, and inflammation, underscore that nicotine is only one of multiple drivers of white matter vulnerability in prenatal tobacco and vaping exposure. Finally, the harm to white matter depends on the cumulative direct and indirect effects of these substances.

### 5.4. Functional Consequences of Tobacco and Vaping Toxicant Exposure on White Matter

The molecular effects exerted by prenatal exposure to tobacco-derived toxins correlate well with functional deficits observed in preclinical models. In rats, prenatal exposure to nicotine (from gestational days 4 to 20) induces at postnatal day 60, impairments in balance and coordination in young offspring [75], both motor functions that depend in part on white-matter integrity [107,108]. As these deficits persist into adulthood at postnatal day 90 [109], these findings also reveal a potential long-lasting prenatal-induced dysfunction in white matter.

**Figure 3:**
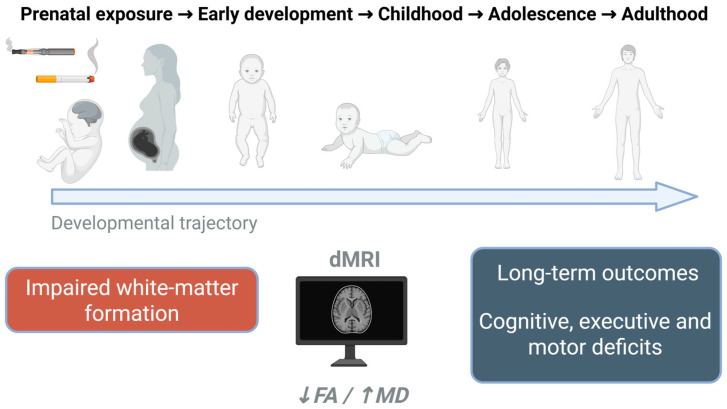
Long-term developmental consequences of prenatal exposure to cigarette smoke and ENDS. Prenatal toxicant exposure disrupts white-matter formation, detectable during early development as decreased fractional anisotropy (FA) and increased mean diffusivity (MD) in dMRI studies. These structural alterations are associated with persistent cognitive, executive, and motor deficits extending through adolescence and adulthood.

Similarly, prenatal exposure to e-cigarette aerosols containing nicotine or to nicotine intake through drinking in pregnant mice promotes behavioral alterations that persist in adult offspring (3 months of age), such as increased locomotor activity [110], impaired object recognition [111] and deficits in attention and working memory. Although these functional impairments do not directly demonstrate white-matter damage, they reflect dysfunction within cortico-striatal, cerebellar, and visuomotor networks that rely in part on intact myelinated pathways for coordination, timing, and sensory integration. Taken together, these experimental data support the view that prenatal exposure to tobacco and vaping toxicants disrupts the maturation of white-matter–dependent circuits, leading to persistent cognitive and sensorimotor inefficiency consistent with the structural and functional alterations observed in human neuroimaging studies (Section 4.2, Figure 3).

## 6. Conclusions and Perspectives

Evidence from epidemiological, neuroimaging, and preclinical studies suggests that tobacco smoke and ENDS aerosols contain multiple toxic substances that may interfere with white-matter development during critical windows of prenatal brain formation. Through mechanisms involving oxidative stress, hypoxia, inflammation, endocrine disruption, and glial metabolic impairment, these toxicants have the potential to alter oligodendrocyte lineage progression and the conditions required for proper myelination in prenatal and early postnatal life. Such alterations have been associated with cognitive and sensorimotor outcomes in some studies, although findings are not yet fully consistent across the literature.

However, the strength and consistency of this evidence varies across studies, and the precise molecular pathways through which these toxicants affect developing myelinated circuits remain incompletely understood. Thus, although alterations in white matter have been reported, the extent to which these changes compromise long-term structural and functional outcomes in humans has not yet been conclusively established. Clarifying the relative contribution of individual toxicants versus combined exposures remains an important challenge for future research.

From a public-health perspective, these findings support the need for preventive strategies aimed at minimizing exposure to toxicants contained in cigarette smoke or vaping aerosols. Likewise, the misconception that prenatal vaping is harmless must be addressed through clear education and regulatory measures.

In summary, integrating epidemiological, imaging, and mechanistic findings provides a coherent framework to understand how prenatal tobacco and vaping exposure may influence white-matter development. Nevertheless, substantial knowledge gaps remain regarding exposure timing, dose-dependence, sex differences, reversibility, and the combined impact of multiple toxicants. Addressing these questions through interdisciplinary research will be essential to develop evidence-based prevention, regulation, and potential therapeutic interventions capable of protecting the developing brain.

## Data Availability

No new data were created or analyzed in this study. Data sharing is not applicable to this article.

## Abbreviations

The following abbreviations are used in this manuscript:

ENDS	Electronic nicotine delivery systems
nAChRs	Nicotinic acetylcholine receptors
OPCs	Oligodendrocyte precursor cells
dMRI	Diffusion magnetic resonance imaging
MRI	Magnetic resonance imaging
FA	Fractional anisotropy
RD	Radial diffusivity
GWC	Gray–white matter contrast
MS	Multiple sclerosis
DHβE	Dihydro-β-erythroidine
VTA	Ventral tegmental area
Bdnf	Brain-derived neurotrophic factor
TrkB	Tropomyosin receptor kinase B
PFC	Prefrontal cortex
CPu	Caudate–putamen
NAc	Nucleus accumbens
Mbp	Myelin basic protein
Mobp	Myelin-associated oligodendrocytic basic protein
Plp1	Proteolipid protein 1
Mag	Myelin-associated glycoprotein
Gje1	Gap junction membrane channel protein epsilon 1
Gjc2	Gap junction protein alpha 12
Cldn11	Claudin11
Ugt8	UDP glycosyltrans-ferase 8
Mal	Myelin and lymphocyte protein
Cnp	2′,3′-cyclic nucleotide 3′-phosphodiesterase
GDNF	Glial cell-derived neurotrophic factor
GFAP	Glial Fibrillary Acidic Protein
Cx43	Connexin-43
CO	Carbon monoxide
NNK	4-(methylnitrosamino)-1-(3-pyridyl)-1-butanone
NRT	Nicotine replacement therapies
